# A Machine Learning Web App to Predict Diabetic Blood Glucose Based on a Basic Noninvasive Health Checkup, Sociodemographic Characteristics, and Dietary Information: Case Study

**DOI:** 10.2196/49113

**Published:** 2023-11-24

**Authors:** Masuda Begum Sampa, Topu Biswas, Md Siddikur Rahman, Nor Hidayati Binti Abdul Aziz, Md Nazmul Hossain, Nor Azlina Ab Aziz

**Affiliations:** 1 Center for Engineering Computational Intelligence Faculty of Engineering and Technology Multimedia University Melaka Malaysia; 2 Department of Computer Science and Engineering Faculty of Science, Engineering and Technology University of Science and Technology Chittagong Chattogram Bangladesh; 3 Department of Statistics Faculty of Science Begum Rokeya University Rangpur Bangladesh; 4 Department of Marketing Faculty of Business Studies University of Dhaka Dhaka Bangladesh

**Keywords:** blood glucose prediction, boosted decision tree regression model, machine learning, noncommunicable diseases, noninvasive

## Abstract

**Background:**

Over the past few decades, diabetes has become a serious public health concern worldwide, particularly in Bangladesh. The advancement of artificial intelligence can be reaped in the prediction of blood glucose levels for better health management. However, the practical validity of machine learning (ML) techniques for predicting health parameters using data from low- and middle-income countries, such as Bangladesh, is very low. Specifically, Bangladesh lacks research using ML techniques to predict blood glucose levels based on basic noninvasive clinical measurements and dietary and sociodemographic information.

**Objective:**

To formulate strategies for public health planning and the control of diabetes, this study aimed to develop a personalized ML model that predicts the blood glucose level of urban corporate workers in Bangladesh.

**Methods:**

Based on the basic noninvasive health checkup test results, dietary information, and sociodemographic characteristics of 271 employees of the Bangladeshi Grameen Bank complex, 5 well-known ML models, namely, linear regression, boosted decision tree regression, neural network, decision forest regression, and Bayesian linear regression, were used to predict blood glucose levels. Continuous blood glucose data were used in this study to train the model, which then used the trained data to predict new blood glucose values.

**Results:**

Boosted decision tree regression demonstrated the greatest predictive performance of all evaluated models (root mean squared error=2.30). This means that, on average, our model’s predicted blood glucose level deviated from the actual blood glucose level by around 2.30 mg/dL. The mean blood glucose value of the population studied was 128.02 mg/dL (SD 56.92), indicating a borderline result for the majority of the samples (normal value: 140 mg/dL). This suggests that the individuals should be monitoring their blood glucose levels regularly.

**Conclusions:**

This ML-enabled web application for blood glucose prediction helps individuals to self-monitor their health condition. The application was developed with communities in remote areas of low- and middle-income countries, such as Bangladesh, in mind. These areas typically lack health facilities and have an insufficient number of qualified doctors and nurses. The web-based application is a simple, practical, and effective solution that can be adopted by the community. Use of the web application can save money on medical expenses, time, and health management expenses. The created system also aids in achieving the Sustainable Development Goals, particularly in ensuring that everyone in the community enjoys good health and well-being and lowering total morbidity and mortality.

## Introduction

Diabetes mellitus (DM), which is characterized by elevated blood glucose levels (BGLs), is a disease that is proliferating swiftly worldwide [1]. The International Diabetes Federation predicted that by the end of 2045, 800 million people would have this disease [2]. Diabetes is a noncommunicable disease (NCD) that is often regarded as the primary cause of numerous illnesses [3]. Uncontrolled diabetes harms almost all body organs. Over time, the excessive glucose in the blood stream causes serious and even mortality-related complications, such as heart conditions, kidney issues, eye issues, diabetic neuropathy, and diabetic retinopathy [4]. Diseases such as asthma, edema, and oral diseases are substantially correlated with diabetes incidence [5].

High blood glucose affects a person’s health and is a risk factor for developing NCDs [6,7]. It is important to detect and monitor diabetes and prevent or control the major complications of the disease. Thus, early prediction of BGLs can reduce the amount of money spent on public health annually and help people become more aware of how to protect themselves from diabetes. Such prediction is crucial to the efficient management of diabetes. The goal of managing type 1 diabetes is to achieve optimal and long-lasting BGL control. This goal can be supported by the automated prediction of BGL using machine learning (ML) techniques, which are thought to be a promising approach. ML is a technique for improving performance by automatically learning from experience and making more accurate predictions [8,9]. Numerous factors influence BGLs. Future BGLs are influenced by prior glucose observations as well as insulin dosage, carbohydrate intake, and other lifestyle factors. Obesity and biomarkers (eg, body fluids including urine, blood, and saliva) are a few of the many factors that have a significant role in the onset and course of DM [9].

In Bangladesh, urban residents exhibit a greater tendency to develop NCDs, including diabetes, than rural residents. This is due to several factors, including their occupational lifestyle (eg, heavy workloads and prolonged sitting to complete their tasks). Thus, people working in the private corporate sector are more likely to develop NCDs [10].

One of the main goals of health policy is to lower the costs associated with controlling NCDs [11]. Hence, studies on how to regularly assess BGLs in a cost-effective way are needed [12]. ML-based prediction models can help to achieve this goal. However, studies supporting the practical validity of ML techniques for predicting health parameters using data from low- and middle-income countries, such as Bangladesh, are very few [13]. Specifically, Bangladesh lacks research using ML techniques to predict BGLs based on basic noninvasive clinical measurements and dietary and sociodemographic information. To formulate strategies for public health planning and the control of diabetes, this study aimed to develop a personalized ML model that predicts the BGLs of urban corporate workers in Bangladesh. We used 5 regression prediction models, including boosted decision tree regression, decision forest regression, Bayesian linear regression, neural network, and linear regression. The models were trained and tested using a data set collected by the team using basic noninvasive clinical measurement devices together with dietary and sociodemographic information. Among the models, boosted decision tree regression exhibited the best performance in predicting the BGLs of the collected data, achieving the lowest root mean square error (RMSE) of 2.3 and mean absolute error (MAE) of 1.3. The model was then incorporated into a web app called the BloodGlucosePrediction Calculator [14], that allows individuals with basic noninvasive health measurements, dietary information, and sociodemographic information to predict their BGL value. Visual Studio was used to develop the web app. The app was developed based on the ML predictive model application programming interface and POST URL. The automated web-based BGL prediction system in this study is capable of aiding communities, especially those in highly remote areas, to diagnose BGLs quickly and easily. 

Many studies on the prediction of BGLs have been conducted, each using a different set of data, patients, and features [15]. For example, a study used recurrent artificial neural networks (ANNs) and Elman recurrent ANNs to predict BGLs based on previous blood glucose values. They used a virtual data set called “Case 002” compiled from the free AIDA simulator. The study used RMSE to measure performance and obtained average RMSE values of 6.43 mg/dL, 7.45 mg/dL, 8.13 mg/dL, and 9.03 mg/dL for prediction horizons of 15 minutes, 30 minutes, 45 minutes, and 60 minutes, respectively. The researchers suggested additional case studies [16].

In another study [17], prediction models using nonlinear autoregressive neural networks and long short-term memory (LSTM) networks were introduced for predicting future glucose levels. These models were trained on a large set of continuous glucose monitoring data. The LSTM model, in particular, demonstrated superior predictive performance, achieving a lower RMSE value of 19.47 compared to other models. In a study using the OhioT1DM data set, which contains data from 6 patients with type 1 diabetes who participated in an institutional review board–approved study for 8 weeks between March 2016 and April 2017, the researchers applied LSTM and neural attention models for blood glucose prediction [18]. Their study found that the LSTM algorithm showed the best performance among the tested models.

The ML model mentioned in the research by Orabi et al [5] was trained on a data set of information from patients with type 2 diabetes. The data set, containing 23 characteristics, was collected by the Egyptian National Research Center specifically from patients with diabetes. The developed system showed 84% prediction accuracy. Meanwhile, Wu et al [19] predicted type 2 DM using the Pima Indian Diabetes data set. They achieved an accuracy of 95.42% using the improved K-means algorithm and the logistic regression algorithm. They suggested further work to develop an application based on the proposed model. Kaur and Kumari [8] also used the Pima Indian Diabetes data set to develop ML models that classify the patients into diabetic and nondiabetic categories [8]. They applied and compared 5 different predictive models. The highest accuracy was reported for the linear kernel support vector machine, which achieved an accuracy of 0.89. The other 4 algorithms, including radial basis kernel support vector machine, k-nearest neighbor, ANN, and multifactor dimensionality reduction, achieved accuracies of 0.84, 0.88, 0.86, and 0.83, respectively.

According to a review of diabetes prediction papers [9], various data set characteristics, such as dimensionality, the number of instances relative to the number of features, or the data set type (genetic or clinical), can significantly impact the algorithm’s performance. As a result, an algorithm that performs best on one data set may have lower prediction accuracy than other algorithms on different data sets.

Numerous studies have focused on predicting BGLs by using various feature sets and applying mathematical and ML models. However, no model has achieved 100% accuracy. Moreover, most of the previous ML-based studies in health care were conducted using data from high-income countries [20]. The application of supervised ML approaches to medical data to predict diseases, the survivability of diseases, and different types of health checkup test results by using sample data from Bangladesh is lacking. A search through several academic databases, including PubMed, IEEE Xplore, and Google Scholar, found that no existing studies specifically focused on ML blood glucose prediction in Bangladesh using dietary and sociodemographic data. Therefore, more research is needed in this area. Consequently, this paper focuses on predicting continuous BGLs based on data from noninvasive basic health checkup tests, dietary information, and sociodemographic characteristics collected from patients from Bangladesh.

## Methods

### Data Collection

The data were collected from the employees at the Grameen Bank complex in Dhaka, Bangladesh, which includes 18 distinct organizations with more than 500 employees, including Grameen Bank, Grameen Communications, other nongovernmental organizations, and private companies. The study collected data from 271 workers to predict their BGLs. Typically, a larger sample size is expected for ML approaches. However, previous studies included relatively few participants (eg, 118 [21] and 300 [22]). It must be mentioned that a small sample size is sometimes associated with higher classification accuracy [23]. A simple random sampling procedure drew the sample. In this study, we informed people first about our portable health clinic (PHC) system. After the initial introduction of the PHC system, the survey was conducted among those who came to the service point and received the PHC service at least once.

We calculated the required sample size using the following sample size formula, S = Z^2^ × P × (1−P)M2, where S is the sample size for infinite population, Z is the *z* score, P is the population proportion (assumed as 50% or 0.5), and M is the margin of error.

Using the above formula for Z=1.960, P=0.5, and M=0.05, we calculated the required sample size to be 384. However, due to missing values, we included 275 observations in our study.

This study collected the test items used as input factors using the human-assisted PHC system. However, measurements such as arrhythmia, blood cholesterol, blood hemoglobin, blood grouping, urinary sugar, and urinary protein were excluded due to many missing cases. The blood glucose measurement was considered as an output factor. The PHC system has been previously described in detail [24].

Clinical measurements were obtained through direct diagnosis using PHC devices operated during data collection by a well-trained nurse or health care professional. The clinical measurements assessed by a PHC were as follows: blood pressure; pulse rate; body temperature; oxygenation of blood; arrhythmia; body mass index (BMI); waist, hip, and waist to hip ratio; blood glucose; blood cholesterol; blood hemoglobin; blood uric acid; blood grouping; urinary sugar; and urinary protein. Data on dietary information and sociodemographic characteristics were collected during face-to-face interviews using a standard questionnaire.

### Regression Predictive Modeling

#### Machine Learning Models

Since the study’s targeted output variable was a continuous variable, a person’s BGL value was predicted using a regression prediction model. A regression predictive model’s performance is reported as an error in those predictions because it predicts a quantity. The most popular ML models, including boosted decision tree regression, decision forest regression, Bayesian linear regression, linear regression, and neural network, were evaluated. This study chose these models for comparison because they are widely used to predict medical data.

#### Linear Regression

The linear regression model is a very simple ML method in which each data point consists of a pair of vectors, namely, the input vector and the output vector. Even on larger data sets, it is the most straightforward, traditional, and widely-applied correlational method [13]. The form of the linear regression model is *U_pred_* = *βx_in_*, where *β* represents the vector of coefficients, which are calculated by applying the least-squares method; *U_pred_* is the predicted value; and *x_in_* is the value of the independent variable [4].

#### Boosted Decision Tree Regression

Boosting is a popular ML ensemble method [25], where boosting in a decision tree ensemble tends to improve accuracy with some small risk of less coverage. It is an ML method for regression issues. It builds each regression tree in a stepwise fashion using a predefined loss function to measure the error in each step and correct for it in the next. Thus, the prediction model is an ensemble of weaker prediction models. In regression problems, boosting builds a series of trees in a stepwise fashion, and then selects the optimal tree using an arbitrary differentiable loss function [26]. Like random forest, it combines many smaller, weaker models into a final summed prediction. However, the idea of boosting is to add new models to the ensemble in a sequence for a number of sequences. In each iteration, a new weak model is trained for the whole ensemble learned up to that new model. These iteratively produced new models are built to be maximally correlated with the negative gradient of the loss function that is also associated with the ensemble as a whole. In this approach, a performance function is placed on the gradient boosting machine to find the point at which adding more iterations becomes negligible in benefit (ie, adding more simple models, in this case decision trees, no longer reduces the error by a significant margin). At this point, the ensemble sums all of the predictions into a final overall prediction [27]. The boosted trees model is an additive model that makes predictions by combining decisions from a sequence of base models. More formally, the class of models can be written as *G*(*x*) = *f*_0_(*x*) + *f*_1_(*x*) + *f*_2_(*x*) + … + *f*_n_(*x*), where the final classifier, *G*, is the sum of simple base classifiers *f_i_*. For the boosted tree model, each base classifier is a simple decision tree. This broad technique of using multiple models to obtain better predictive performance is called model ensembling.

#### Neural Network

The neural network is a network of connected neurons. The neurons cannot operate without other neurons with whom they are connected. Usually, they are grouped in layers, process data in each layer, and pass it forward to the next layer. The last layer of neurons makes the decisions [28].

#### Decision Forest Regression

This regression model consists of an ensemble of decision trees. A collection of trees constitutes a forest. Each tree in a regression decision forest outputs a Gaussian distribution as a prediction. Aggregation is performed over the ensemble of trees to find a Gaussian distribution closest to the combined distribution for all trees in the model [29]. This technique generates several decision trees during training, which can split randomly from a seed point. This results in a “forest” of randomly generated decision trees whose outcomes are ensembled by the random forest algorithm to predict more accurately than a single tree does alone. One problem with a single decision tree is overfitting, which makes the prediction seem very good on the training data but unreliable in future predictions [27]. By using decision forest regression, a model can be trained with a relatively small number of samples and produce good results.

#### Bayesian Linear Regression

Recently, Bayesian learning has been widely adopted and proven more powerful than other ML techniques. Bayesian linear regression allows a fairly natural mechanism to survive insufficient or poorly distributed data. It allows putting a prior on the coefficients and noise so that the priors can take over in the absence of data. The result of Bayesian linear regression is a distribution of possible model parameters based on the data and the prior. This allows the uncertainty about the model to be quantified with fewer data points, and the posterior distribution will be more spread out.

### Experimental Setup

**Figure 1:**
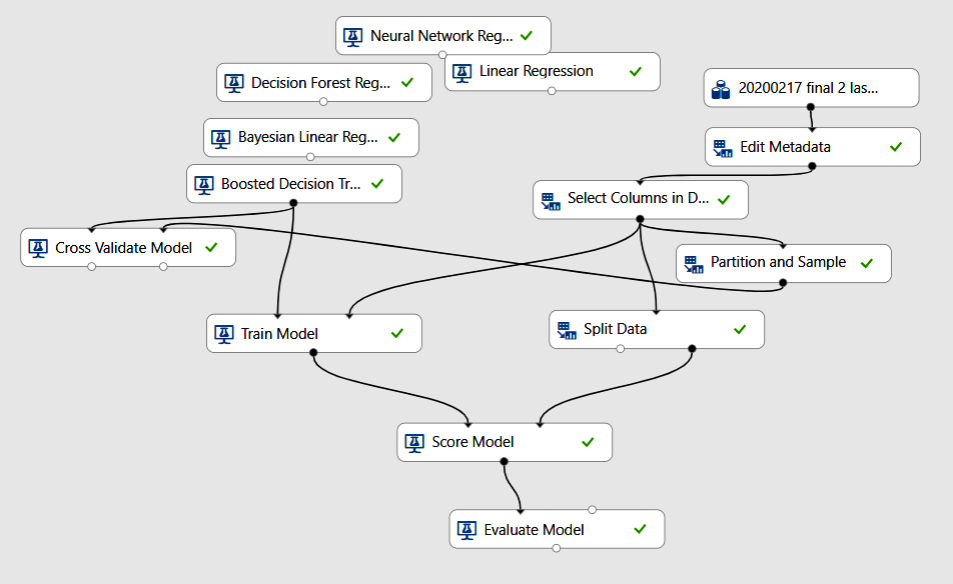
Flowchart of Azure machine learning algorithms.

Azure Machine Learning Studio (Microsoft Corp) was used for the implementation of trained models. Azure Machine Learning Studio provides a user-friendly, visual approach to building ML pipelines. Azure Machine Learning Studio offers a graphical interface that allows users to design and build ML pipelines using a drag-and-drop approach. It also provides a wide range of prebuilt modules for data preprocessing, feature engineering, model training, and evaluation. These modules can be combined to create complex pipelines without writing code. We trained 5 relevant ML models using our data. The detailed technical aspects of the modeling process are shown in Figure 1.

Figure 1 shows the ML algorithms used in this study. First, the data file was uploaded into the experiment. Then, the data were edited, and variables for use in the predictive algorithms were selected. Next, the data set was split, and the ML algorithms were trained with the given data set to develop the model. After training, the score model was obtained and the trained model was evaluated to measure its accuracy (ie, performance).

To evaluate the performance of the models, RMSE values from each model were used. The RMSE of a model is the average difference between the model’s prediction and the actual outcome [27]. It indicates how close the predicted value is to the actual value. There is no cutoff or benchmark for RMSE values. The smaller the value, the better the prediction. The MAE was also used. It is the sum of the absolute differences between predictions and actual values. Additionally, the coefficient of determination (*R*^2^) was measured. *R*^2^ represents how close the predicted value is to the actual value, and a higher value of *R*^2^ is desirable.

Each model was trained on a 70% training sample to ensure uniform training. We split data according using a 0.7:0.3 training set to test set ratio. We did not use the cross-validation method because K-fold cross-validation produces strongly biased performance estimates with small sample sizes [23].

**Figure 2:**
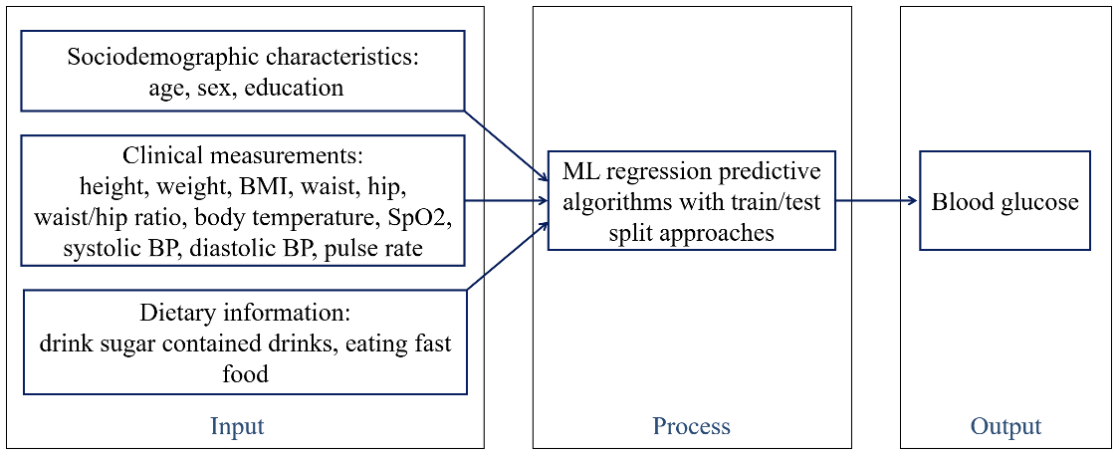
The input-process-output model used to predict blood glucose levels after processing 17 input variables using ML algorithms. BMI: body mass index; BP: blood pressure; ML: machine learning.

The input-process-output model for predicting blood glucose based on sociodemographic characteristics, dietary information, and some basic health checkup test results is shown in Figure 2. The 3 types of inputs were fed into the process using the 5 ML regression algorithms. The output was the predicted BGL.

### Ethical Considerations

The authors obtained ethics approval from the National Research Ethics Committee of the Bangladesh Medical Research Council (18325022019).

## Results

### Description of the Study Population

The descriptive statistics of the study participants and the summary statistics of the selected categorical predictors used in ML are shown in Table 1 and Table 2, respectively.

The mean age of the respondents was 49.61 (SD 7.39) years, and most participants were aged 50 years. On average, the respondents had a BMI of 25.37 (SD 3.20). The World Health Organization defines BMIs ranging from 25 to 29.9 as overweight; therefore, most of the respondents were overweight. The respondents’ average BGL was on the borderline at 128.02 (SD 56.92) mg/dL (whereas the normal value is 140 mg/dL). This shows that it is important for the respondents to check their blood glucose regularly.

The majority (n=225, 83%) of the respondents were men and most had completed at least a college or university degree. Among the 271 respondents, 9.6% (n=26) reported that they drink sugar-containing drinks (eg, Coke, Fanta, soda, or fruit juice) 3 or more times a week, and 18.1% (n=49) reported that they eat fast foods (eg, pizza, hamburgers, and deep-fried foods) 3 or more time a week.

**Table 1:** Summary statistics of the selected continuous predictors used in machine learning (n=271).

Variables	Range	Mean (SD)
Age (years)	34-77	49.61 (7.39)
Height (cm)	140.00-184.00	163.05 (7.45)
Weight (kg)	44.20-114.40	67.52 (10.06)
BMI^a^ (kg/m^2^)	18.39-40.53	25.37 (3.20)
Waist (cm)	63.60-118.00	90.24 (7.80)
Hip (cm)	80.00-127.00	94.54 (6.29)
Waist:hip ratio	0.64-1.11	0.96 (0.06)
Body temperature (°F)	92.12-99.64	96.07 (1.15)
SpO2^b^	93-99	97.67 (1.17)
Systolic BP^c^ (mmHg)	92-180	126.68 (14.88)
Diastolic BP (mmHg)	59-108	81.71 (8.43)
Pulse rate (bpm)	51-123	80.27 (11.66)
Blood uric acid	3.10-11.00	6.63 (1.54)
Blood glucose (mg/dL)	66.60-392.40	128.02 (56.92)

^a^BMI: body mass index.

^b^Blood oxygenation.

^c^BP: blood pressure.

**Table 2:** Summary statistics of the selected categorical predictors used in machine learning.

Category		Participants (n=271), n (%)
**Gender**		
	Men	225 (83)
	Women	46 (17)
**Education**		
	No education (no school entered)	10 (3.7)
	Primary school completed	30 (11.1)
	Secondary school completed	11 (4.1)
	High school completed	23 (8.5)
	Vocation school completed	1 (0.4)
	College or university completed	63 (23.2)
	Higher education (master or doctor) completed	133 (49.1)
**Drinks sugar-containing drinks^a^**		
	Yes	26 (9.6)
	No	245 (90.4)
**Eats fast foods^b^**		
	Yes	49 (18.1)
	No	222 (81.9)

^a^Drinks sugar-containing drinks (eg, Coke, Fanta, soda, fruit juice, orsother Sweet or sugar-containing drinks) 3 or more times a week.

^b^Eats fast foods, such as pizza, hamburgers, or deep-fried foods (eg, singara, samosa, moglai parata) 3 or more times a week.

### Prediction Performance Assessment

To examine the prediction performance of the regression predictive technique using ML, the main evaluation criterion used was the RMSE. The RMSE measures the average magnitude of the error by taking the square root of the average of the squared differences between the prediction and actual observations. The RMSE value gives us an idea of how much, on average, the predicted values deviate from the actual values; it is a measure of the fit of the model to the data points. Table 3 presents the performance of the regression models. The boosted decision tree regression model performed the best among the tested models. Its RMSE was the lowest at a value of 2.30, indicating that, on average, the boosted decision tree regression model’s predicted blood glucose deviated from the actual blood glucose by around 2.30 mg/dL. Its RMSE and MAE were significantly lower than those of the other models, while it’s *R*^2^ was the largest (0.99).

**Figure 3:**
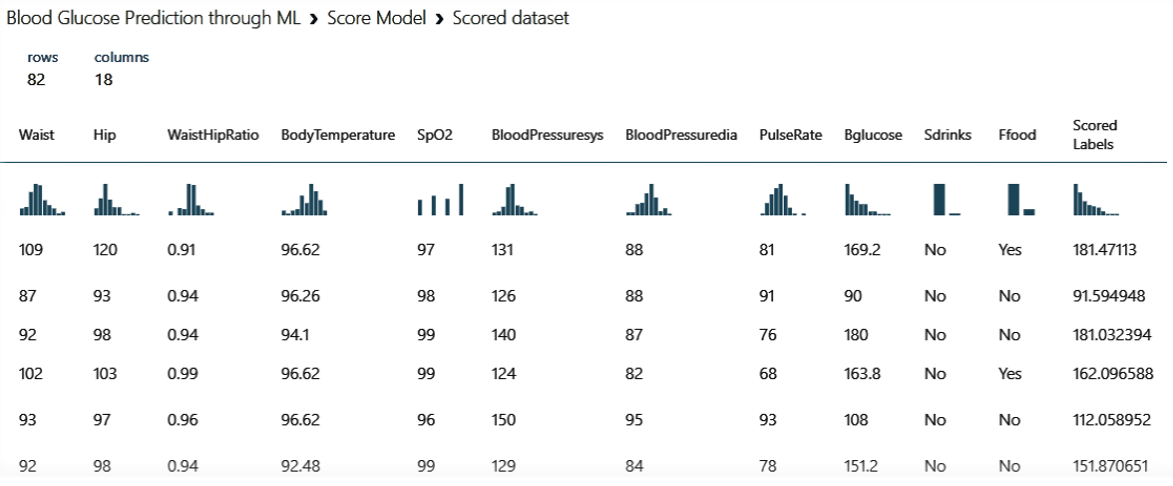
Partial view of the score model (prediction of blood glucose level) obtained by the boosted decision tree regression model. BloodPressuresys: systolic blood pressure; BloodPressuredia: diastolic blood pressure; Bglucose: blood glucose level; Ffood: fast food; Sdrinks: sugary drinks.

The score model of the best predictive model—the boosted decision tree regression—is shown in Figure 3. The “Scored Labels” column (last column) in Figure 3 indicates the predicted value of BGLs. It can be seen that the deviation between the scored labels and the actual BGL reported in the “Bglucose” column was small.

**Table 3:** Comparison of modeling techniques.

Model name	Root mean squared error	Mean absolute error	*R* ^2^
Neural network	45.54	36.24	0.18
Decision forest regression	22.99	17.46	0.79
Linear regression	44.66	35.59	0.21
Boosted decision tree regression	2.30	1.30	0.99
Bayesian linear regression	44.49	34.50	0.22

## Discussion

### Principal Findings

ML algorithms can identify the patterns in a data set that may not be directly apparent. Thus, ML can provide helpful information and support to medical staff by identifying patterns that may not be obvious [30]. Comparison of the results found in this study to those in previous related works is crucial. Most previous studies have reported performance measurements as a function of classification accuracy, which may not be directly comparable to this study, which adopts a regression approach to build a predictive model for the continuous variable (ie, BGL).

A previous study compared the performance of 3 ML models, namely logistic regression, ANN, and decision tree models, for predicting diabetes or prediabetes using 12 common risk factors (including only 1 clinical factor: BMI) in Guangzhou, China [31], and found accuracies of 77.87%, 73.23%, and 76.13% for the decision tree model, ANN, and logistic regression model, respectively.

The gradient boosting ML algorithm has been successfully used in clinical studies to predict cardiovascular diseases [12]. The gradient boosting decision tree method by Friedman [32] predicted BMI with an accuracy of 0.91 [33]. In the current study, decision tree regression was the best predictive model, followed by decision forest regression. Both of these are ensemble learning methods.

**Figure 4:**
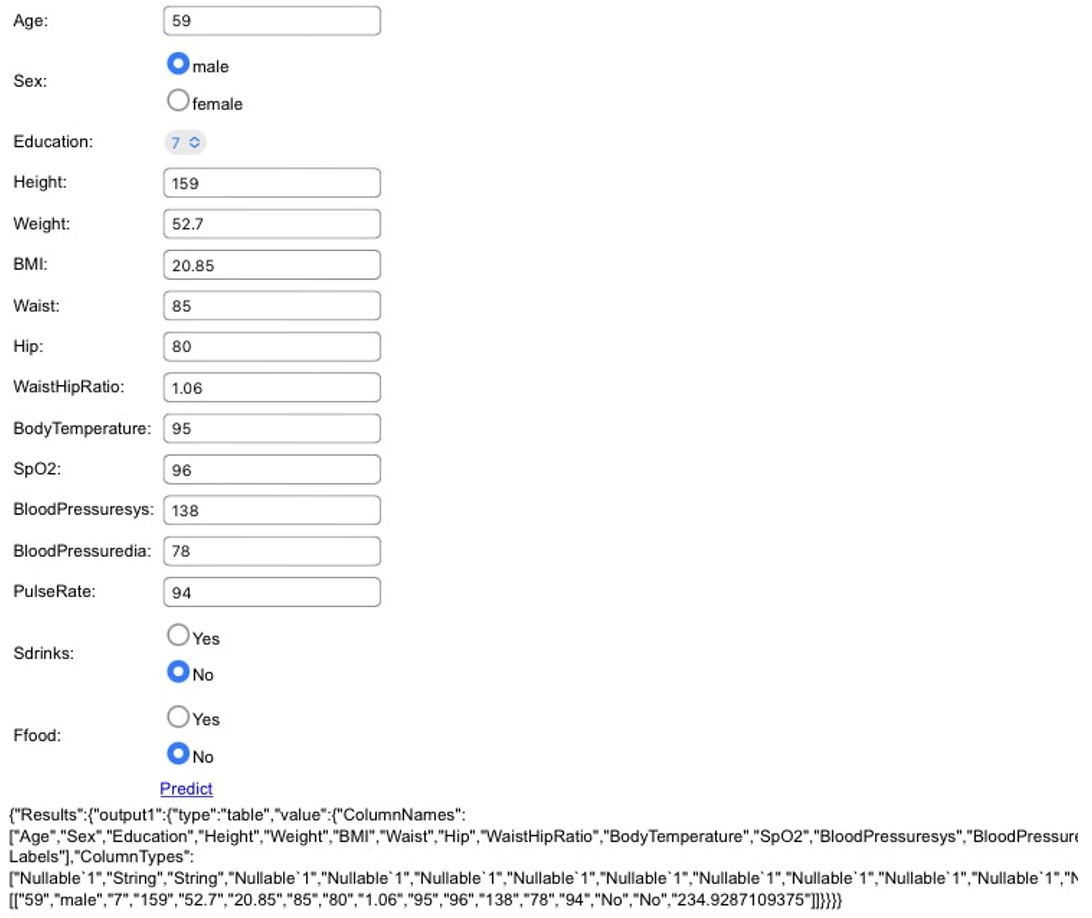
Screenshot view of the website to predict blood glucose value obtained by integrating the best performing boosted decision tree regression model into the application. BloodPressuresys: systolic blood pressure; BloodPressuredia: diastolic blood pressure; Bglucose: blood glucose level; Ffood: fast food; Sdrinks: sugary drinks.

In this study, 5 ML prediction algorithms were evaluated to predict blood glucose values. The health records of 271 employees aged 34 to 77 years were collected and used in this study. The data included well-known relevant factors of high blood glucose, such as age and BMI, as well as other factors with unknown associations with BGL. Specifically, 17 noninvasive health measurements, dietary information, and sociodemographic information were used for the prediction. This study used portable, cheap, and affordable devices for clinical data collection. The low-input dimensions provided the advantage of keeping the necessary time to train the models relatively small. Blood glucose was set as an output factor. Among the studied prediction algorithms, boosted decision tree regression was found to be the most effective with the best RMSE (2.3); this RMSE value was better than any reported in the literature. The developed model is a powerful tool for predicting blood glucose with limited medical resources and has been deployed on a website [14]. Figure 4 shows the web app. With this app, the patients of caregivers do not need to carry the blood glucose measuring instruments or be concerned with ensuring enough supply of test strips and lancet needles, as the predictor is able to predict BGL using external factors.

### Conclusions

This study provides a measure for reducing NCDs and can be a good component in the national or global plan. We developed a blood glucose prediction model based on personal characteristics, dietary information, and some basic clinical measurements related to NCDs. Such a blood glucose prediction model is useful for reducing health management costs and improving awareness among high-risk individuals. Predicted glucose values can be used for early hypoglycemic or hyperglycemic alarms. These predictions can also help prevent complications associated with high BGLs. As a result, the developed model can help achieve the Sustainable Development Goals, ensuring universal and equal health coverage, and thus reducing overall morbidity and mortality.

However, there are several limitations to this study. First, the number of samples we studied was small; it must be expanded to train the prediction model in the future. Second, this study was limited to a particular area, specifically among employees working in a corporate office. We collected the training data from this specific area. We trained our model by using these data only. The data from other institutes do not confirm our prediction model. Our findings may not be applicable to a broader population or different geographical locations. This is because the characteristics, behaviors, and dynamics of the population might differ significantly from one area to another. As a result, it becomes challenging to generalize the results beyond the confines of the chosen area and employee group. The specific area and employee group might possess unique cultural, economic, or contextual factors influencing their behaviors and responses. These factors might not hold true for other settings or groups, leading to skewed conclusions if applied outside the study’s scope. The data from other institutes do not confirm our prediction model. However, the framework achieves high performance on Grameen Bank complex data. Thus, we believe this data-based strategy is also fit for predicting blood glucose in other communities. In future studies, additional features (eg, work stress, everyday physical activity, eating red meat) should be considered to improve the prediction accuracy. Nonetheless, this study served as a successful case.

## Abbreviations

ANN: artificial neural network
BGL: blood glucose level
BMI: body mass index
DM: diabetes mellitus
LSTM: long short-term memory
MAE: mean absolute error
ML: machine learning
NCD: noncommunicable disease
PHC: portable health clinic
RMSE: root mean square error
